# Position of Joe Biden’s Administration on the World Trade Organization

**DOI:** 10.1134/S1019331622120061

**Published:** 2022-09-29

**Authors:** A. M. Menshikova

**Affiliations:** Institute for US and Canadian Studies, Russian Academy of Sciences, Moscow, Russia

**Keywords:** WTO, Joe Biden’s administration, economic policy, trade policy, WTO reform, liberal trade, protectionism, EU, China

## Abstract

The election of Democratic Party candidate Joe Biden as President of the United States did not change Washington’s negative attitude towards the activities of the World Trade Organization (WTO). The historically established consensus of the Republican and Democratic parties, expressed in the general similarity of the approach of the legislative and executive branches of the U.S. government to the WTO as a tool primarily for realizing U.S. national interests in the foreign economic sphere, hinders the achievement of generally acceptable agreements within the WTO in key areas of its activities. Like the previous administration of Donald Trump, Joe Biden’s administration has, in particular, been blocking the activities of the WTO’s Appellate Body for a number of years. Despite the Democratic President’s statements about the commitment of the United States to the principles of liberal trade, the White House, as before, proceeds from the desire to maintain the leading role of the United States in the WTO, even at the cost of curtailing certain areas of its work. The dominant desire is to transform the WTO into an international economic mechanism to strategically contain China and openly oppose Russia by politicizing the WTO and taking measures that pave the way for the complete dismantling of the rules-based multilateral trading system. The WTO is in fact in a state of permanent institutional crisis in a number of central areas of its activity. The only way to deal with the current crisis is to give economics precedence, not politics, and prevent violations of agreed multilateral trade rules by unilateral actions; otherwise the negative impact on world markets and the economies of many WTO members will continue.

## INTRODUCTION

The United States of America, being one of the founders of the World Trade Organization (WTO) in 1995, has recently been the most severe critic of the multilateral trading system. At the time of the creation of the WTO, the United States was one of the three largest trading countries in the world. American companies continue to benefit enormously from the trade rules that are created, controlled, and enforced through the WTO. No other member of the organization has used the organization’s dispute resolution mechanism as frequently as the United States to address potential violations by other member states.^1^ However, there are several aspects that formed the overall critical attitude of official Washington towards the activities of this international organization even before Donald Trump took office as president [Menshikova, 2021].

## CONSENSUS OF REPUBLICANS AND DEMOCRATS IN WTO ASSESSMENT

There is a consensus in Washington between the views of the Republican and Democratic parties that the WTO does not meet the requirements of the changed world. It is believed that the WTO’s negotiations failed to update the rules of international trade related to the impact of nonmarket economic factors and unfair trade practices: forced technology transfer and massive industrial subsidies. The impact of new technologies, such as the Internet, has not been taken into account. The obligations of member countries in key areas related to free trade agreements, primarily related to intellectual property and the service sector, have not been revised. It was not possible to significantly reduce or equalize the tariff regime between large economies.

WTO negotiations did not result in new rules or additional opportunities for market access, the system for monitoring compliance with the requirements and obligations of members of the organization did not hold countries responsible for ignoring the basic principles, and the dispute settlement system did not strictly apply the rules agreed in the form as they were originally announced. The WTO is accused of members of the organization lack the necessary consensus related to the acceleration of the processes of opening up national economies in emerging market countries. The White House virtually excludes the possibility of ensuring fair international competition through the WTO, bearing in mind the trade and economic rivalry between the United States and China. The US government, starting with the administration of George Bush Jr. was particularly dissatisfied with the WTO’s arbitration system, where the United States regularly lost trade disputes when other countries took action against American antidumping practices. The WTO’s appeals body has been accused of exceeding the organization’s original mandate, and its activities have been characterized as infringing on US sovereignty: “The Appellate Body has regularly made rulings that have made it difficult for countries to fight unfair trade practices and protect jobs,” said United States Trade Representative D. Robert Lighthizer in Donald Trump’s administration [1]. As a result, the WTO Appellate Body effectively ceased to function in December 2019 due the United States blocking the procedure for appointing the original number of judges.

Washington criticized the excessively long decision-making procedures. The administrations of B. Obama and D. Trump prevented the nomination of new judges to the WTO arbitration courts. In the fall of 2020, the US blocked the appointment of a new CEO of the organization, and as a result, the process remained frozen until the US presidential election in November. The Republican administration of Donald Trump prevented the coordination of the processes of large-scale reform of the organization, showing interest only in the reform of the rules of transparency, electronic commerce, and the Agreement on Subsidies and Compensatory Measures. De facto bypassing the system of settlement of international trade disputes in the WTO, President Trump applied protectionist tariff regulations on hundreds of billions of dollars worth of imports from China, the EU and many other countries by resorting to US national security legislation. Making the goal of his foreign economic strategy the strengthening of US sovereignty over trade policy and the revision of international trade agreements, Donald Trump, especially during the COVID-19 pandemic took a clear course so that the United States could leave the WTO, which to a large extent deepened the fundamental crisis. Moreover, the negative attitude was not limited to the US leadership.

Farmers and wage labor organizations criticized the WTO for focusing too much on corporate interests. Environmentalists spoke out against the organization’s decisions on genetically modified foods and what the organization considers discriminatory eco-labels. Experts have argued that the Intellectual Property Agreement—“Trade Aspects of Intellectual Property Rights”—and the WTO’s drug patent rules restrict access to medicines in the world’s poorer countries, and that WTO rules abolish national sovereignty and thereby undermine environmental protection and labor. The unions say the organization is not effective in protecting US wages from undermining unfair labor practices abroad, arguing, for example, that some countries are violating the basic rights of workers in the developing world to adequate wages. This approach is reflected in the low cost of their products in comparison with similar industries in industrialized countries. Developing countries respond that attempts to review labor standards in the WTO are a form of protectionism in disguise. Some economists say that by encouraging imports and moving operations overseas, WTO-led tariff cuts are hurting US jobs and wages. “The WTO no longer guarantees access to mandatory, two-tier, independent and impartial trade dispute resolution. This is a clear violation of the WTO’s agreements,” the European Union stated [2].

The WTO’s Appellate Body still does not have the quorum needed to hear appeals because President Trump’s administration, insisting that the WTO had exceeded its mandate, blocked the appointment of new candidates in December 2019, effectively depriving the organization of its ability to resolve international trade disputes. Unilateral tariffs imposed under the pretext of national or economic security requirements undermine the credibility of the WTO and its key rules and principles and lead to new trade restrictions, as was the case with the US trade policy under Donald Trump on China.

At the December 2018 summit, the G20 leaders endorsed the following wording in their statement: “International trade and investment are important engines of growth, productivity, innovation, job creation and development. We recognize the contribution that the multilateral trading system has made to these goals. At present, the system is not achieving its goals and there is room for improvement. Therefore, we support the necessary reform of the WTO to improve its functioning.” [3].

## APPROACH OF JOE BIDEN’S ADMINISTRATION TO INSTITUTIONAL REFORM OF THE WTO

The Democratic administration of President Biden, declaring its commitment to openness in the activities of multilateral organizations and readiness for negotiation processes in US trade policy, focused on the national interests of the country, confirmed the need for reform of the WTO. It was declared to be useful as an “effective tuning tool to restore the relevance of the global trade body to the workers,” while maintaining the traditional leadership role of the United States in the organization’s activities. At the same time, as part of the overall strategic course towards the virtual rejection of the principle of unconditional free trade, the need was emphasized to identify and rethink aspects of the existing trading system that stimulate or allow so-called unfair competition. The WTO’s “shortcomings” such as its “cumbersome and bureaucratic” decision-making processes, its existence in a “bubble” isolated from reality, excessively slow recognition of global events [4], when “the reality of the institution today does not match the ambitions of its goals” [5], and dispute resolution has become “synonymous with litigation,” which is “lengthy, costly and contentious,” were publicly criticized. It has been stated that too often the rules of global trade are designed to provide benefits that are not based on fair competition or American values more broadly [6].

In February 2021, WTO members approved Ngozi Okonjo-Iweala of Nigeria, the first woman and first African to lead the organization as CEO. Joe Biden announced her appointment two weeks after his inauguration as U.S. president. However, in principle, the US administration’s position in relation to the WTO indicates an orientation towards a fundamental change in general international trade relations: from the Ricardian ideal of free trade to mutual protectionism in a world where the United States, Europe, and other world economic centers are fighting for geo-economic dominance with the help of subsidies, tariffs, nontariff trade barriers, technological decoupling, and purely national industrial policy. Washington has, above all, taken a strategic lesson from the pandemic-driven situation in global supply chains, especially in the chip industry: value chains for future technologies must be shortened and new manufacturing facilities for key industries must be created exclusively in the United States itself.

The WTO is the main governing body of international trade and acts as a negotiating forum, arbiter, and observer for the implementation of trade agreements. However, in recent years, major WTO negotiations have stalled, with many countries turning to bilateral or regional free trade agreements to advance their trade interests. Negotiations on a comprehensive development agenda failed over disagreements over agricultural subsidies and intellectual property rights.

The Biden’s administration emphasized its commitment to the organization, but largely continued the approach of adopted by the Republicans under President Trump in its relations with the WTO; in particular, it allowed the blocking of the appointment of new judges to the WTO Appellate Body to continue. This allows countries against which complaints have been filed to indefinitely ignore rulings against them, while their appeal is pending. A group of two dozen countries, as well as the European Union, were forced in this situation to create an alternative arbitration system to resolve disputes in the interim. The US reaction to this was negative. In response to the Appeal Bodies’ proposed appointments, the U.S. administration state it was “unable” to uphold the decision because “the United States continues to have systemic problems with the appellate body. As the members know, the United States has been raising and explaining its systemic issues for over 16 years and through several US administrations [7] . “Over the years, the Appellate Body has overstepped its authority and misinterpreted WTO agreements in a number of cases to the detriment of the United States and other WTO members. In addition, the Appellate Body did not follow existing rules designed to resolve disputes in a timely manner. Reforms are needed to ensure that the root causes of such problems do not come to the surface and that the Appellate Body does not diminish the rights and obligations of WTO members” [8]. President Biden’s administration is convinced that the Appellate Body in its current form threatens the ability of the United States to protect itself from unfair trade practices in a competitive global economy.

An analysis of the latest data from the US presidential administration on the recent activities carried out by the United States in the WTO allows us to single out the main ones, which, in fact, are not of fundamental importance for the implementation of large-scale institutional reforms of the organization. The United States took the following steps in the WTO in 2021 [9]:

• in the Committee on Agriculture, together with Canada, the European Union, and Japan, a formal proposal was presented to achieve greater transparency in agriculture, and, together with Canada, Chile, Colombia, Paraguay and Uruguay, a technical paper was prepared on the public storage of stocks for food security;

• the Rules Negotiating Group continued to play a leading role in achieving results and advocating for strict rules on subsidies for fisheries, and put forward a proposal that the results of the negotiations could contribute to the efforts of WTO members to address the problems of forced labor on fishing vessels;

• the Dispute Settlement Body put forward proposals to improve the transparency and efficiency of WTO dispute settlement and called on its members to consider the rules for submissions *amicus curiae*, that is, appeals from persons who are not parties to the dispute;

• at the Council for Trade in Services, as in previous years, at the request of the United States, discussions continued on the cybersecurity measures of China and Vietnam, in terms of potentially adverse effects on trade. The US continued to express concern about certain measures taken by the Russian Federation regarding software preinstallation mandates and some tax incentives offered to Russian software and information technology companies;

• in the Internal Regulations Working Group, the United States decided to participate in negotiations on the text of rules regarding authorization requirements and procedures for service providers, as well as technical standards for services;

• the US reiterated its unwillingness to agree to launch a process to fill vacancies in the WTO Appellate Body, thereby allowing it to continue hearing appeals without the participation of WTO members and in resolving these critical issues;

• the US Committee on Trade and the Environment worked to promote priorities related to trade in recyclable and reclaimable materials and focused members on post-consumer “reverse supply chains” to reduce barriers to trade and support resource efficiency in production models. In November 2021, the United States formally joined the informal dialogue “Structured Discussions on Trade and Environmental Sustainability” and cosponsored a ministerial statement outlining dialogue’s priorities for 2022;

• in 2020–2021, the United States continued to provide technical assistance to Afghanistan, Georgia, Jordan, Kazakhstan, the Lao People’s Democratic Republic, the Republic of Moldova, Ukraine, and Vietnam to meet their membership obligations;

• since 2021, the Office of the US Trade Representative has become actively involved in the Trade and Gender Informal Working Group program (established in September 2020 to advance efforts to increase women’s participation in global trade). In July 2021, the office hosted a presentation by the US Department of Commerce’s Commercial Law Development Program on national programs to build capacity for the economic empowerment of women through commercial and economic reforms.

## OUTLOOK FOR THE EVOLUTION OF THE UNITED STATES’ WTO POLICY

Despite the change in tone on the WTO, there are few signs that the United States will prioritize addressing key issues related to a truly massive reform of the organization. The long-standing, historically typical for Democrats, negative attitude towards the WTO, as well as significant disagreements between the United States and its key allies, including the EU, on the issues of institutional reform of the WTO, remains in place. In particular, the US and the EU hold opposing views on the WTO’s dispute settlement system and whether it is acceptable for a WTO court to set rules and improve the base of common international law without the consensus of all members of the organization. The United States rejects this idea, while the EU as a whole accepts it.

The concerns raised by the United States go beyond the Appellate Body and cover essentially all the core functions of the WTO. Both Republicans and Democrats have expressed dissatisfaction with the issue of “emerging country status”: the ability of large emerging countries (India) to independently claim their status as an “emerging country” in order to maintain the preferences of such status and avoid the same obligations as states-competitors with developed economies.

From a fundamental point of view, the core of the generally negative approach to the WTO reflects a common, long-established bipartisan consensus in Washington of both the legislative and executive branches of government regarding the policy on a principled policy of strategic containment of China, which, in relation to the activities of the WTO, is expressed in the thesis of the “inability” of the organization to counter China’s “bad faith” trade policy and bring it under concerted multilateral pressure, which portrays this policy as a threat to the global world trading system as a whole. The United States argues that the WTO rules were not designed to effectively address the problems of emerging markets such as China, which are not fully fledged market economies. Such sentiments intensified after a WTO arbitration in January 2022 stated China could retaliate against $645 million in annual US exports in a decade-long trade dispute over US anti-subsidy duties on Chinese goods. The amount disclosed was far less than the $2.4 billion initially requested by China when it filed the case in 2012, when China complained to the WTO that the US had imposed illegal countervailing duties on a dozen Chinese imports, including thermal paper, pipes, citric acid, lawn mowers, kitchen shelving, magnesia bricks, printed graphics, solar panels, wind towers, and steel sinks. The American side argued that the decision was “deeply disappointing” and “reflects misinterpretations by the Appellate Body that damage the ability of WTO members to protect our workers and businesses from China’s trade-distorting subsidies <…> the decision reinforces the need to reform WTO rules and resolve disputes that used to protect China’s nonmarket economic practices and undermine fair, market-oriented competition” [10].

## CONCLUSIONS

President Biden’s administration is basing it work at the WTO on the premise that the organization provides a forum solely for enforcing US rights under various WTO agreements to ensure that the United States receives all the benefits of membership. It is clearly stated that WTO members must rethink the approach to development within this organization, and that the time has come to move beyond the outdated Doha Development Agenda. To maintain its status as a viable institution and fulfill the functions of its original mandate, the WTO must focus its work on structural reform, find the means to achieve trade liberalization between ministerial conferences, and adapt to address the challenges faced by traders today.

In the future, however, there is no evidence of the readiness of the US administration to significantly contribute to the institutional reform of the WTO in the interests of all participants in international trade. In contrast, a policy of politicizing the activities of this organization, which is clearly contrary to the fundamental principles of the WTO, has been outlined, initiated by the collective West under the leadership of the United States and having nothing in common with the initially declared goals and principles. The Russian Federation circulated a statement among WTO members, in which it drew attention to the danger to the international trading system due to the politicization of trade and the introduction by a number of countries of restrictions on trade with the Russian Federation that violate WTO rules. “The Russian Federation would like to draw the attention of WTO members to the dangers looming over the multilateral trading system due to recent aggressive and politically motivated trade restrictive actions by some members. Instead of encouraging the gradual normalization of international trade that is needed for the economy to recover from the pandemic, these members are gradually implementing unilateral trade measures aimed at undermining the economies of Russia and its neighbors. Recently, the reckless "economic war” unleashed by these members has escalated to a critical point, resulting in “collateral damage around the world,” the Russian statement noted [11].

## Footnotes

1 As of January 2022, a total of 607 cases had been initiated at the WTO. This includes the cases filed by the United States as a plaintiff (124, as a defendant (156), and as a third party (171). During the presidency of Barack Obama, from 2009 to 2017, the United States filed 25 cases, more than any other country during this period. Sixteen of them were aimed at China. The United States won seven cases against China, including cases challenging Chinese agricultural and aviation subsidies and import duties on steel.
